# Influence of Solution Deposition Process on Modulating Majority Charge Carrier Type and Quality of Perovskite Thin Films for Solar Cells

**DOI:** 10.3390/ma12152494

**Published:** 2019-08-06

**Authors:** Chuangchuang Chang, Xiaoping Zou, Jin Cheng, Tao Ling, Yujun Yao, Dan Chen

**Affiliations:** 1 Beijing Advanced Innovation Center for Materials Genome Engineering, Research Center for Sensor Technology, Beijing Key Laboratory for Sensor, MOE Key Laboratory for Modern Measurement and Control Technology, School of Applied Sciences, Beijing Information Science and Technology University, Jianxiangqiao Campus, Beijing 100101, China; 2 State Key Laboratory on Integrated Optoelectronics, Institute of Semiconductors, Center of Materials Science and Optoelectronics Engineering, University of Chinese Academy of Sciences, Chinese Academy of Sciences, Beijing 100864, China

**Keywords:** PSCs, one-step, two-step, perovskite thin film, P type, N type, homojunction, solution deposition

## Abstract

In the past ten years, extensive research has witnessed the rapid development of perovskite solar cells (PSCs) and diversified preparation processing craft. At present, the most widely used methods of preparing perovskite solar cells are the one-step method and the two-step method. The main work of this paper is to study the effect of the solution deposition process on the quality of perovskite thin films, as well as modulating majority charge carrier types. Perovskite film was prepared in air by designing different processes, which were then adequately analyzed with corresponding methods. It was demonstrated that the preparation process plays a crucial role in modulating the type of majority carrier and in achieving high-quality perovskite thin film. The one-step prepared perovskite layer is enriched in MA^+^, leading to a P type majority carrier type thin film. The two-step prepared perovskite layer is enriched in Pb^2+^, leading to a N type majority carrier type thin film. In addition, we found that the one-step method caused PbI_2_ residue due to component segregation, which seriously affects the interface and film quality of the perovskite layer. This work aims to modulate the majority carrier type of perovskite film through different preparation processes, which can lay the foundation for the study of homojunction perovskite solar cells to improve the device performance of PSCs.

## 1. Introduction

The perovskite solar cell (PSC) technology has developed rapidly over the past decade. Despite toxicity and stability problems [1,2], PSCs have still attracted much attention due to low cost, simple preparation, and excellent photoelectric properties [3,4,5,6,7]. In 2009, the efficiency of PSC prepared by Kojima et al. is 3.8% [8]. Recently, the efficiency of PSC has reached to 25.2%, according to the latest certification report from the National Renewable Energy Laboratory [9]. Nowadays, solution deposition techniques are widely used in the preparation of PSCs devices. Different preparation techniques contribute to changing many electronic properties, such as carrier concentration, majority carrier type, carrier mobility and electrical resistivity [10,11,12,13]. In previous studies, heterojunctions were prepared between the perovskite layer and the carrier transport layer using a one-step process or two-step process. However, the perovskite layer itself also can be self-doped into a P type or N type to form a homojunction [14,15,16,17]. The homojunction inside the perovskite layer can form a built-in electric field to increase the migration ability of electrons and holes, thereby reducing carriers recombination in the perovskite film layer [14]. Therefore, understanding the elements of the solution deposition method is crucial in predicting its majority carrier type for the rational design of PSCs devices. The most widely used methods are the one-step solution deposition and the two-step solution deposition [18]. However, which, the one-step or the two-step method, can improve the performance of PSCs? What are the specific differences between the two?

In 2013, the Gratzel group adopted the two-step continuous deposition method, which broke the previous method of together dissolving spin-coating of PbI_2_ and CH_3_NH_3_I (MAI) in previous experimental studies, which improved the efficiency of the PSCs [19]. In 2014, Im et al. conducted a comparative study on the one-step process method and the two-step process method. The paper pointed out that the coverage of perovskite in TiO_2_ layer would have a significant impact on device performance, and concluded that the two-step process is superior to the one-step process [20]. In 2018, Li et al. conducted a comparative analysis on the preparation of inverted PSCs using the one-step and the two-step processes under different preparation conditions [21]. These papers [21,22,23] focused on the influence of different preparation environments on the device performance, and pointed out the influence of PbI_2_ residue. In the same year, Wang et al. conducted quantitative analysis to study the effects of different low-temperature processes in the glove box on the electrical properties of perovskite thin films, and concluded that the two-step method had more potential [18].

Although different solution deposition methods have been studied in previous studies, no one has studied the effects of the one-step and the two-step methods on the majority carrier type of perovskite films. In the past, the majority carrier type of perovskite thin film was changed by self-doping or extrinsic doping, but it was found that the preparation of perovskite film by different processes can also change the majority carrier type. In previous studies, Li et al found that the majority carrier type of perovskite films can be regulated between the P type and the N type via controlling growth conditions, but no evidence was supplied on how the preparation process impacts the majority carrier type of perovskite films so that the performance of PSCs devices is affected [14]. The majority carrier type of perovskite film not only affects the location of the heterojunction, but also provides data for the study of homojunction PSCs. Therefore, it is necessary to study the effects of different solution deposition processes on the preparation of perovskite thin films.

Herein, we envisage whether it is possible to regulate the majority carrier type of perovskite film by different solution deposition processes. This experiment was carried out in air, high temperature conventional PSCs were prepared via the one-step process method and the two-step process method under the same environmental conditions. In this article, we supplied the experimental data that CH_3_NH_3_PbI_3_ can be either P or N self-doped using different preparation processes of perovskite. The majority carrier types of perovskite films were found to be regulated by the film composition that was strongly influenced by the preparation process, precursor composition, and process conditions. Due to different processes causing segregation of perovskite layer components, the perovskite layer is rich in Pb^2+^ or rich in MA^+^, and the result is self-doping into N type or P type perovskite film [24]. Our experimental results show that we can modulate the majority carrier type of perovskite film by controlling the preparation process. The P-type perovskite film is prepared by a one-step method, and the N-type perovskite film is prepared by a two-step method. Thus, this study will be helpful in making the performance of PSCs better.

## 2. Materials and Experimental Methods

### 2.1. Materials

Fluorine-doped SnO_2_ (FTO) as substrate of perovskite thin films was purchased from Shanghai MaterWin New Materials Co., Ltd. (Shanghai, China). The N, N-dimethyl formamide (DMF) and the Dimethyl sulfoxide (DMSO) were purchased from Alfa Aesar (China) Corp. (Shanghai, China). The isopropanol (IPA) and the chlorobenzene, the acid titanium dioxide solution (bl-TiO_2_) and the 18NR-T TiO_2_ (mp-TiO_2_) were purchased from Shanghai MaterWin New Materials Corp. (Shanghai, China). The Methylammonium iodide (MAI) and the Spiro-OMeTAD solution, the PbI_2_ and the NaI were purchased from Xi’an Polymer Light Technology Corp. (Xi’an, China). The gold-plated electrode metal was produced by Beijing Zhongjinyan New Material Technology Co., Ltd. (Beijing, China).

### 2.2. Device Fabrication

**Figure 1:**
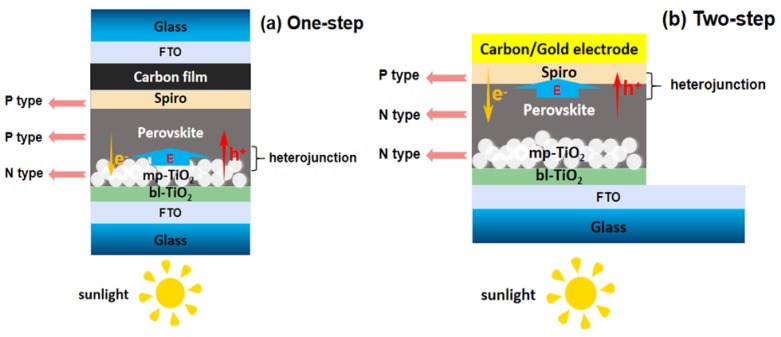
The structure of the device prepared by the one-step method (**a**) and two-step method (**b**) from top to bottom are fluorine-doped SnO_2_ FTO (C/Au)/Spiro-OMeTAD/CH_3_NH_3_PbI_3_/mp-TiO_2_/bl-TiO_2_/FTO. The figure includes the direction of the built-in electric field, the direction of carrier migration and the majority carrier type of perovskite films.

Figure 1 displays the structure of the device which prepared the one-step solution deposition (a) and the two-step solution deposition (b) from top to bottom are FTO (C/Au)/Spiro-OMeTAD/CH3NH3PbI3/mp-TiO_2_/bl-TiO_2_/FTO. As the photoelectric anode material of solar cell devices, FTO conductive glass must be cleaned carefully before use. After cutting and separating the conductive glass, an ultrasonic device was used to sequentially clean the conductive glass with a mixed solution (detergent and deionized water), glass water (acetone: deionized water: 2-propanol = 1:1:1) and alcohol in sequence. After ultrasonic cleaning, rinsed three times with deionized water and dried for 30 min. It was then treated with ultraviolet ozone (UVO) for 1 h before use to increase hydrophilicity. Next, acid TiO_x_ solution was rotated and coated at 2000 rpm for 60 s to prepare the dense layer of TiO_2_ (bl-TiO_2_), and then heated by hot plate at 100 °C for 10 min for heat treatment. After heat treatment, the substrates were put into the muffle furnace and calcined at 500 °C for 30 min to obtain the intact TiO_2_ dense layer. Next, 18NR-T TiO_2_ slurry solution was rotated and coated at 2000 rpm for 60 s to prepare the TiO_2_ mesoporous layer (mp-TiO_2_), and then heated by hot plate at 100 °C for 10 min for heat treatment. Finally, the substrates were put into the muffle furnace and calcined at 500 °C for 1 h to obtain the intact TiO_2_ mesoporous layer.

**Figure 2:**
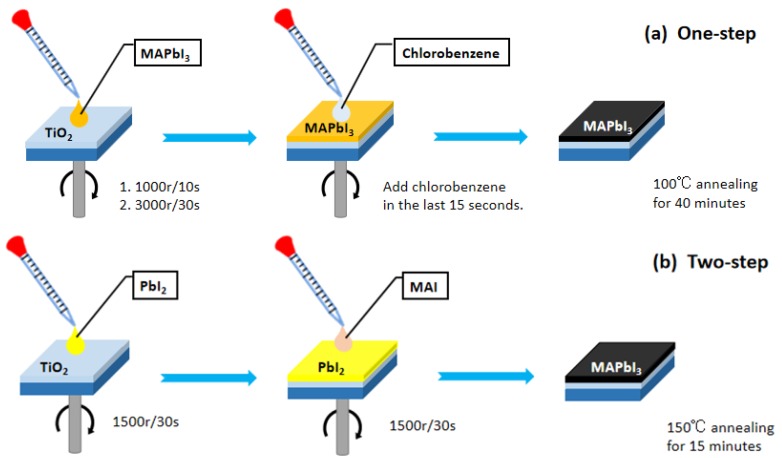
Flow chart of preparation of perovskite film by the one-step (**a**) method and the two-step (**b**) method.

Figure 2a is the one-step preparation of perovskite film; 0.5993 g PbI_2_ (1.3 mol/L) was weighed with an electronic balance and baked on a hot plate at 70 °C for 30 min to remove moisture, PbI_2_ changed from light yellow to orange. Then, 0.1908 g MAI (1.2 mol/L) was weighed and mixed with PbI_2_. Two kinds of precursor PbI_2_ and CH_3_NH_3_PbI (MAI) were dissolved in the mixed solution of N, N-dimethylformamide (DMF) and dimethyl sulfoxide (DMSO) (volume ratio of 4:1). In order to make the drug dissolve evenly, the agent was put into the ultrasonic equipment for ultrasonic dissolution for 15 min. Next, it was stood for 12 h, and the agent was filtered before use. The preparation of the perovskite layer had two successive spin coating stages including the first stage of 1000 rpm for 10 s, and the second stage of 3000 rpm for 30 s. Twenty microliters of chlorobenzene were added with 15 s left in the second stage. Perovskite thin film was obtained after 40 min of heat annealing with a 100 °C hot plate. Spin-coating spiro was used on the perovskite film at 3000 rpm for 30 s to prepare a hole transport layer. Finally, the counter electrode was conductive glass as the substrate, and the carbon film was formed by the smoke particles generated by the burning of the candle. The prepared carbon film was aligned on the top layer of the prepared device and the side was clamped with a butterfly clip for easy packaging.

Figure 2b is the two-step preparation of perovskite film; 0.5993 g PbI_2_ (1.3 mol/L) was weighed with an electronic balance and baked on a hot plate at 70 °C for 30 min to remove moisture, PbI_2_ changed from light yellow to orange. Following this, the precursor of PbI_2_ was dissolved in the mixed solution of N, N-dimethylformamide (DMF) and dimethyl sulfoxide (DMSO) (volume ratio of 0.95:0.05). Placed in an ultrasonic device for sonication until the solute was completely dissolved, then filtered solution. Next, 0.07 g MAI was weighed with an electronic balance, and 1 mL of isopropanol (solvent) was added. It was placed in an ultrasonic device for sonication until the solute was completely dissolved, and then filtered. The first step of the two-step method was to spin-coat the PbI_2_ solution at 1500 rpm for 30 s on mesoporous TiO_2_ substrate. The second step of the two-step method was to spin-coat the MAI solution on PbI_2_ substrate at 1500 rpm for 30 s, and place it on the hot plate heat annealing at 150 °C for 15 min. The perovskite thin film was then obtained. Spin-coating spiro on the perovskite film was used at 3000 rpm for 30 s to prepare a hole transport layer. Finally, the counter electrode was conductive glass as the substrate, and the carbon film was formed by the smoke particles generated by the burning of the candle. The prepared carbon film was aligned on the top layer of the prepared device and the side was clamped with a butterfly clip for easy packaging. The device was also prepared by a two-step gold-plated electrode, and an electron transport layer, a perovskite layer and a hole transport layer were formed on the etched glass substrate. Finally, it was put into the vapor deposition chamber and vapor-deposited on the gold electrode.

The Hall effect test described in this paper is a perovskite film prepared on a glass substrate. In other tests, perovskite film refers to the film prepared on carrier transport layer. All the tests contain substrates. The perovskite layer described refers to a single layer of perovskite, and the test results do not include other layers.

### 2.3. Characterization

The morphology details of perovskite films were measured by scanning electron microscope (SEM) (SIGMA, Zeiss, Jena, Germany). Energy dispersive X-ray spectroscopy (EDS) was used for testing and analyzing the content of chemical constituents in perovskite films. X-ray diffraction (XRD) data from samples of perovskite thin films substrates were collected using an X-ray diffractometer (D8 Focus, Bruker, Dresden, Germany). The absorption spectra of perovskite films with different solution deposition process step were analyzed using an ultraviolet (UV) visible absorption spectrometer (Avantes, Apeldoom, The Netherlands) and the Photoluminescence Spectroscopy (PL) data were obtained by a LabRAW HR800 PL testing system (HORIBA JObin Yvon, Paris, France). Hall effect data were measured by the Hall Effect Measurement System HL5500PC (QUATEK, Shanghai, China). The photocurrent density-voltage (J-V) characteristics were measured under simulated standard air quality daylight (AM 1.5, 100 mW/cm^2^) with a solar simulator (Sol 3A, Oriel, New Port, RI, USA).

## 3. Results and Discussion

**Figure 3:**
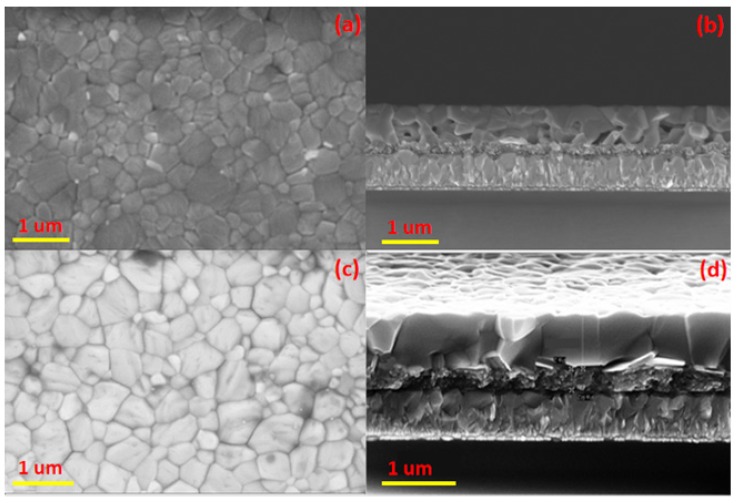
Top view and cross-sectional view of the SEM images for one-step (**a**,**b**) and two-step (**c**,**d**).

Figure 3a,b portrays the top view and cross section of perovskite thin film prepared by the one-step method, Figure 3c,d portrays the top view and cross section of perovskite thin film prepared by the two-step method. It can be clearly seen from the top view that the one-step process and the two-step process prepared perovskite layers are flat, but the two-step prepared perovskite has a larger grain size. In the cross-section Figure 3d, it can be noticed that the upper surface of the perovskite film prepared via the one-step method is flat, but the lower surface (the interface in contact with the mesoporous layer) has many holes. The formation of these pores may be related to the dropping time and the amount of dropping of chlorobenzene. The added chlorobenzene did not come to the lower surface of the perovskite, so that the crystallinity of the lower surface was not good, and pores appeared. It can also be observed from the Figure 3b that the perovskite grains prepared via the one-step method do not penetrate the entire perovskite layer, and there are many perovskite grain boundaries in the vertical direction of the perovskite film. On the contrary, the perovskite film prepared via the two-step process method has fewer grain boundaries in the vertical direction and the perovskite grains almost vertically penetrate across the entire perovskite layer, which is an important reason for higher conversion efficiency of the perovskite photoelectric device by using the two-step preparation. It can be concluded that the interface of the perovskite film prepared via the two-step process method is better than that of the one-step prepared perovskite film, which may have a crucial influence on the ability of the perovskite layer carrier to be injected into the titanium dioxide layer.

**Figure 4:**
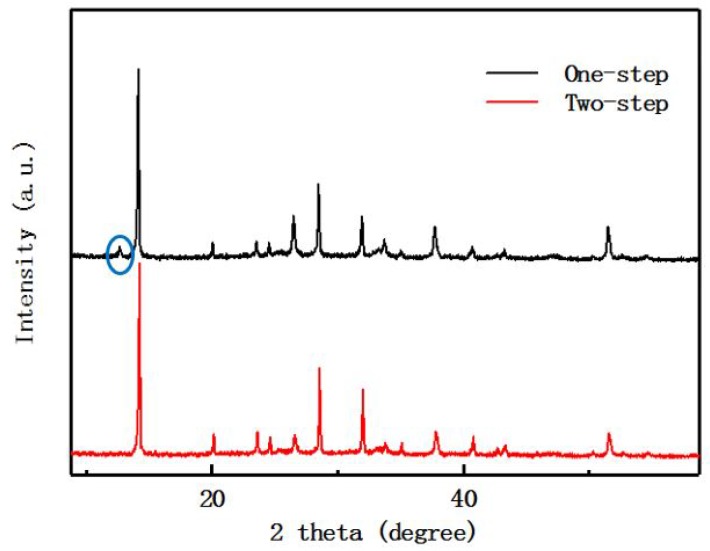
XRD patterns of perovskite films with one-step and two-step. The blue circle is the peak of residual PbI_2_.

Figure 4 is the XRD comparison of the preparation of perovskite film via the one-step process method and the two-step process method. The blue circle in Figure 4 is the peak of residual PbI_2_. It can be seen from the XRD pattern that the XRD peak position of the perovskite prepared via the one-step process method and the two-step process method are almost the same. Besides, the peak of PbI_2_ is present in the XRD image of the one-step prepared perovskite film, which illustrates that residue of lead PbI_2_ in the perovskite film prepared via the one-step process method. However, the strength of the perovskite XRD peak prepared by the two processes is different. The change of the XRD peak of the perovskite show that there is no obviously difference in the crystal structure of the perovskite prepared by the two methods, which can be considered as the same crystal structure type [25]. The residue of PbI_2_ has an impact on the interface of the perovskite film, which affects the efficiency of the device [16,17,18].

**Figure 5:**
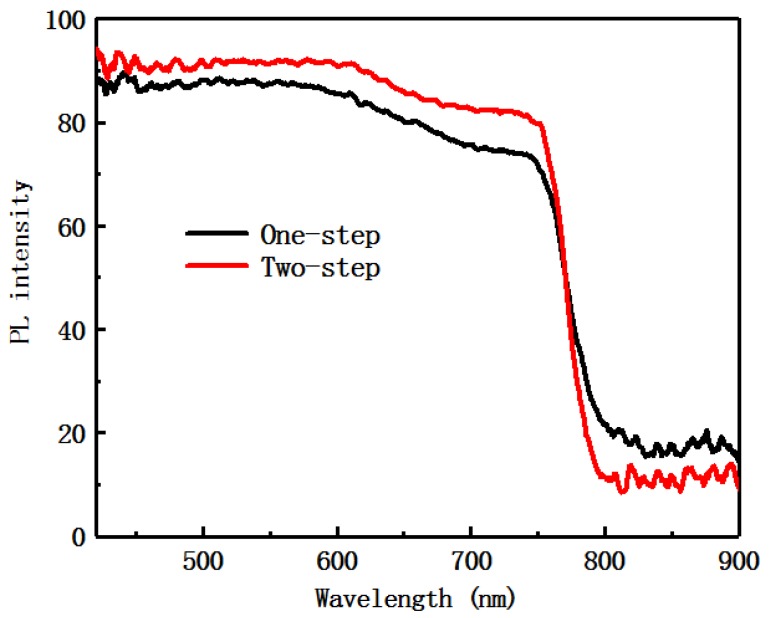
UV-visible absorption spectra of perovskite films with the one-step and the two-step methods.

Figure 5 is a comparison of the ultraviolet absorption spectra of the samples prepared via the one-step and the two-step methods. It can be observed from the Figure 5 that most of the light absorption wavelength is distributed in the visible range, and the effective absorption intensity of the two-step sample is higher than that of the one-step sample. It is indicated that the absorbance of the sample prepared via the two-step process method is stronger than that of the sample prepared via the one-step process method, and the absorption band side shows a blue shift.

**Figure 6:**
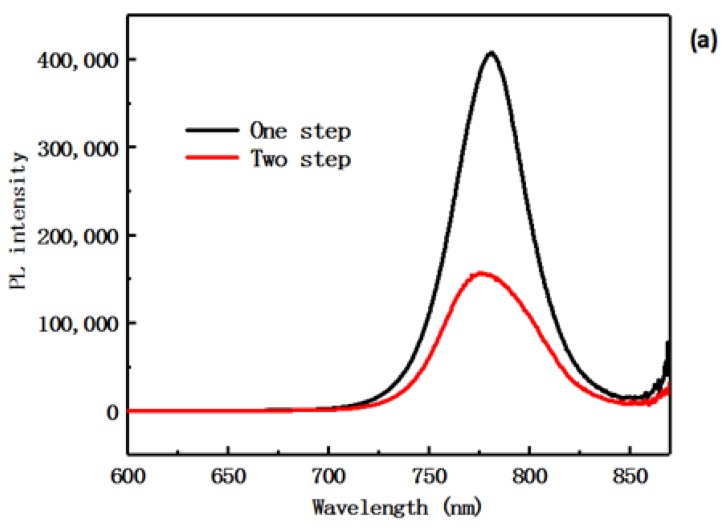

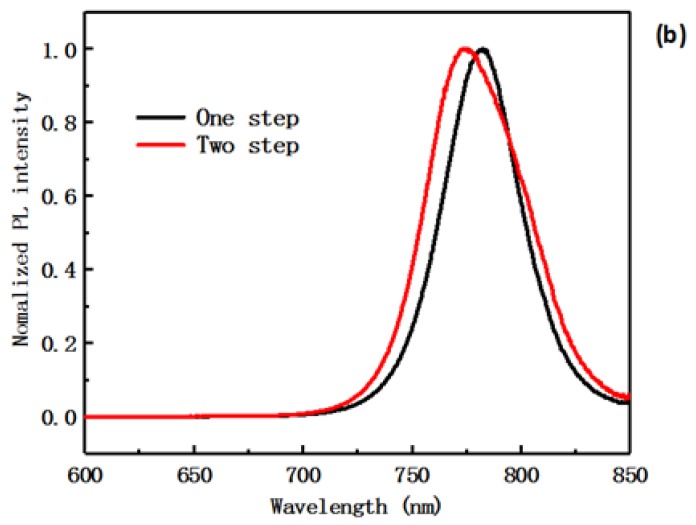
Photoluminescence (PL) spectra of perovskite films with the one-step process method (**a**) and the two-step process method (**b**).

Figure 6a,b show photoluminescence (PL) spectra of samples prepared via the one-step and the two-step methods. The experiments in this paper were all perovskite layers prepared on the TiO_2_ layer. In Figure 6a, the strength of the emission peak of the sample prepared via the two-step process method is lower than that of the sample prepared via the one-step process method, indicating that the ability of the two-step prepared perovskite film carrier to inject the TiO_2_ layer is stronger. The SEM experiments indicate that the interface and film quality of the two-step process method sample are better than that of the one-step process method sample, which is consistent with the experimental results obtained in Figure 6a. In Figure 6b is a normalized PL spectrum, in which the curve of the two-step sample exhibits a blue shift compared to the curve of the one-step sample. This is consistent with the displacement of the absorption band edge in the ultraviolet absorption spectrum of Figure 5.

In this paper, the Hall effect was tested on the one-step sample and the two-step sample. In Table 1, the one-step perovskite film of the sample tested was a P type semiconductor, and the two-step perovskite film was an N type semiconductor. The P type semiconductor is a majority of hole carriers, and the N type semiconductor is a majority of electron carriers. Annealing temperature and annealing time will affect the physical properties of the crystal, and may also be related to the process, because the two-step method will be left for a few seconds after the first step of the spin coating PbI_2_. In this paper, the one-step method is 100 °C, annealing for 40 min, and the two-step method is 150 °C, annealing for 15 min. It is indicated that the different solution deposition process methods of the one-step and the two-step will produce segregation of components, which have a great effect on the crystal of the perovskite thin film and may change the photophysical semiconductor characteristics of the film. In the XRD comparison chart of Figure 4, the residue of PbI_2_ was found in the one-step perovskite thin film. From this we can infer the one-step prepared perovskite film is rich in MAI, so the perovskite film is MA^+^ is rich and Pb^2+^ is deficient. The Hall-effect semiconductor type measured in the one-step sample of this experiment is P type consistent with the paper [20].

**Figure 7:**
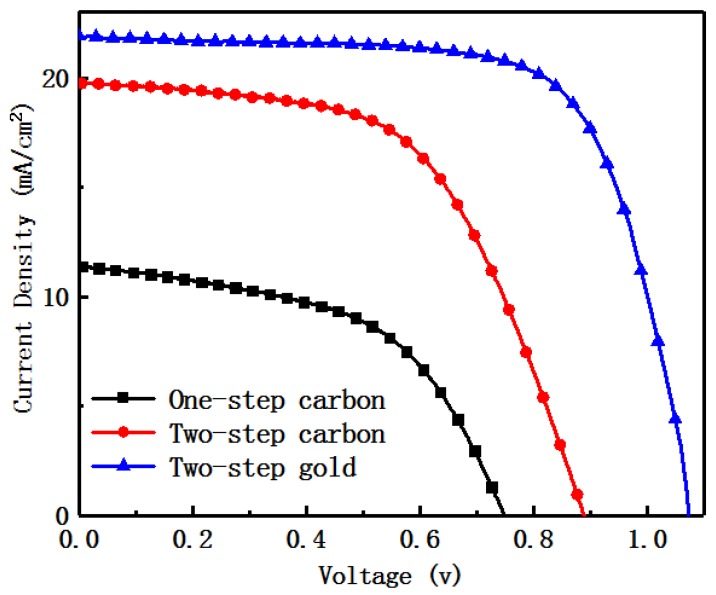
Photocurrent voltage density reverse scan (RS) curve of One-step carbon, Two-step carbon and Two-step gold.

Table 2 shows the photovoltaic parameters of the carbon film perovskite device prepared via the one-step method and two-step method, and the gold electrode perovskite device prepared by the two-step method. Combined with the J-V curve of Figure 7, there can be seen that the open circuit voltage, current density and the fill factor of the two-step prepared perovskite device are higher than those of the one-step preparation device, and the current density and device efficiency of the device prepared via the two-step process method are almost twice as large as the one-step process method, which shows that the two-step method has obvious advantages compared with the one-step method. Mainly due to the great difference in the perovskite films prepared by the two different processes, combined with the SEM, XRD, UV absorption and PL spectra of the experimental samples, the quality of perovskite films prepared via two-step method is obviously higher than those prepared via the one-step method. 

In addition, the gold electrode perovskite device with the two-step method was added in this experiment. Compared with the two-step carbon film electrode, the open circuit voltage and current density of the two-step gold electrode device increased. The impact factor increased from 0.57 to 0.7, and the device efficiency increased from 10.94% to 14.66%, both of which improved greatly.

## 4. Conclusions

The one-step process compared to the two-step process method, the one-step process method requires the counter-solvent chlorobenzene to be dropped. The anti-solvent addition time and amount have a great influence on the quality of the perovskite thin film, and the requirements of the operator are extremely high, with less repeatability. Through the combination of SEM image, XRD pattern and PL spectrum analyses, it was found that the surface of the perovskite thin film prepared by the one-step process method has PbI_2_ residue, so that the ability of carriers to inject TiO_2_ in the perovskite layer is poor. In comparison, the interface of the perovskite film prepared by the two-step method is better than that of the one-step prepared perovskite film. Due to the interface of the perovskite layer prepared by the two-step method, the film quality is better and the carrier injection into TiO_2_ is stronger. Combined with the comparison of device efficiencies, it is concluded that the two-step process is better for the preparation of perovskite devices.

It was found that the one-step prepared perovskite layer was rich in MA^+^, and the Pb^2+^ deficiency made the film appear as a P type semiconductor. The two-step prepared perovskite layer was rich in Pb^2+^, and the MA^+^ deficiency makes the film appear as a N type semiconductor. It can be said that we have found that the electronic properties of the perovskite film, such as the carrier concentration, mobility, majority carrier type, could be significantly tuned by changing their preparation process. This work laid the foundation for future research on homojunction perovskite solar cells. 

## Figures and Tables

**Table 1:** Hall effect parameters with one-step and two-step methods.

Process ^a^	No.	Rs ^b^ (ohm/sq)	Mob ^c^(cm²/Vs)	N ^d^(/cm³)	Type ^e^
One-step Method	1	1.4 × 10^9^	520	+1.8 × 10^11^	P
One-step Method	2	9.8 × 10^8^	680	+1.9 × 10^11^	P
Two-step Method	1	4.7 × 10^9^	494	−5.4 × 10^10^	N
Two-step Method	2	6.7 × 10^9^	598	−3.1 × 10^10^	N

Notes: a. Process method; b. Surface resistivity; c. Hall mobility; d. Carrier concentration; e. Majority carrier type.

**Table 2:** Photovoltaic detailed parameters of perovskite devices prepared by one-step and two-step methods.

Samples	V_oc_^a^ (V)	J_sc_^b^ (mA/cm^2^)	FF ^c^ (%)	PCE ^d^ (%)
One-step carbon	0.75	11.37	0.52	4.43
Two-step carbon	0.98	19.73	0.57	10.94
Two-step gold	1.07	22.26	0.70	14.66

Notes: a. Open-circuit voltage; b. Short-circuit photocurrent density; c. Fill factor; d. Power Conversion Efficiency.

## Data Availability

Data in this manuscript is available.
